# Supplementary material for: An optimized Nurr1 agonist provides disease-modifying effects in Parkinson’s disease models
Source: Nat Commun. 2023 Jul 18;14:4283. doi: 10.1038/s41467-023-39970-9 (PMC10354055; doi:10.1038/s41467-023-39970-9)
Supplement: Supplementary file 1 — Supplementary Information [file 41467_2023_39970_MOESM1_ESM.pdf]

Supplementary Information

An optimized Nurr1 agonist provides disease-modifying effects in Parkinson's disease models

Woori Kim, Mohit Tripathi, Chunhyung Kim, Satyapavan Vardhineni, Young Cha, Shamseer Kulangara Kandi, Melissa Feitosa, Rohit Kholiya, Eric Sah, Anuj Thakur, Yehan Kim, Sanghyeok Ko, Kaiya Bhatia, Sunny Manohar, Youngbin Kong, Gagandeep Sindhu, Yoon-Seong Kim, Bruce Cohen, Diwan S Rawat, Kwang-Soo Kim

List of Supplementary Figures

Supplementary Fig. 1. 4A7C-301 is brain-penetrant and potently activates Nurr1's transcriptional and neuroprotective function

Supplementary Fig. 2. Effect of 4A7C-301 and other Nurr1 agonists on the transcriptional activities of mutant Nurr1-LBD constructs in SK-N-BE(2)C cells

Supplementary Fig. 3. 4A7C-301 exhibits highly potent transactivation and neuroprotection compared to CQ

Supplementary Fig. 4. Environmental (MPP^+) and genetic (αSyn) risk factors of PD compromise the level of Nurr1 but not those of other key transcription factors in MN9D cells

Supplementary Fig. 5. Effects of CQ and 4A3C-301 on dopaminergic gene expression in MN9D cells and mouse ventral mesencephalic (VM) primary cells

Supplementary Fig. 6. Comparison of CQ and 4A7C-301 for their suppressive function of LPS-induced *TNFα* gene expression in BV2 cells

Supplementary Fig. 7. Altered autophagy process by CQ but not by 4A7C-301 in HeLa cell

Supplementary Fig. 8. Assessment of motor and non-motor behaviors at the chronic stage (D14-D15) of MPTP-induced mice

Supplementary Fig. 9. Recovery of motor and non-motor deficits by CQ and 4A7C-301 in AAV-αSyn-induced PD model mice

Supplementary Fig. 10. Restoration of reduced DA levels by CQ and 4A7C-301 in MPTP- and αSyn-induced mouse models

List of Supplementary Tables

Supplementary Table 1. Structure modification information, EC50 and maximal activity of top 36 compounds selected from > 570 4A7C-derivatives

Supplementary Table 2. Primer sequences for site-directed mutagenesis for generation of constructs

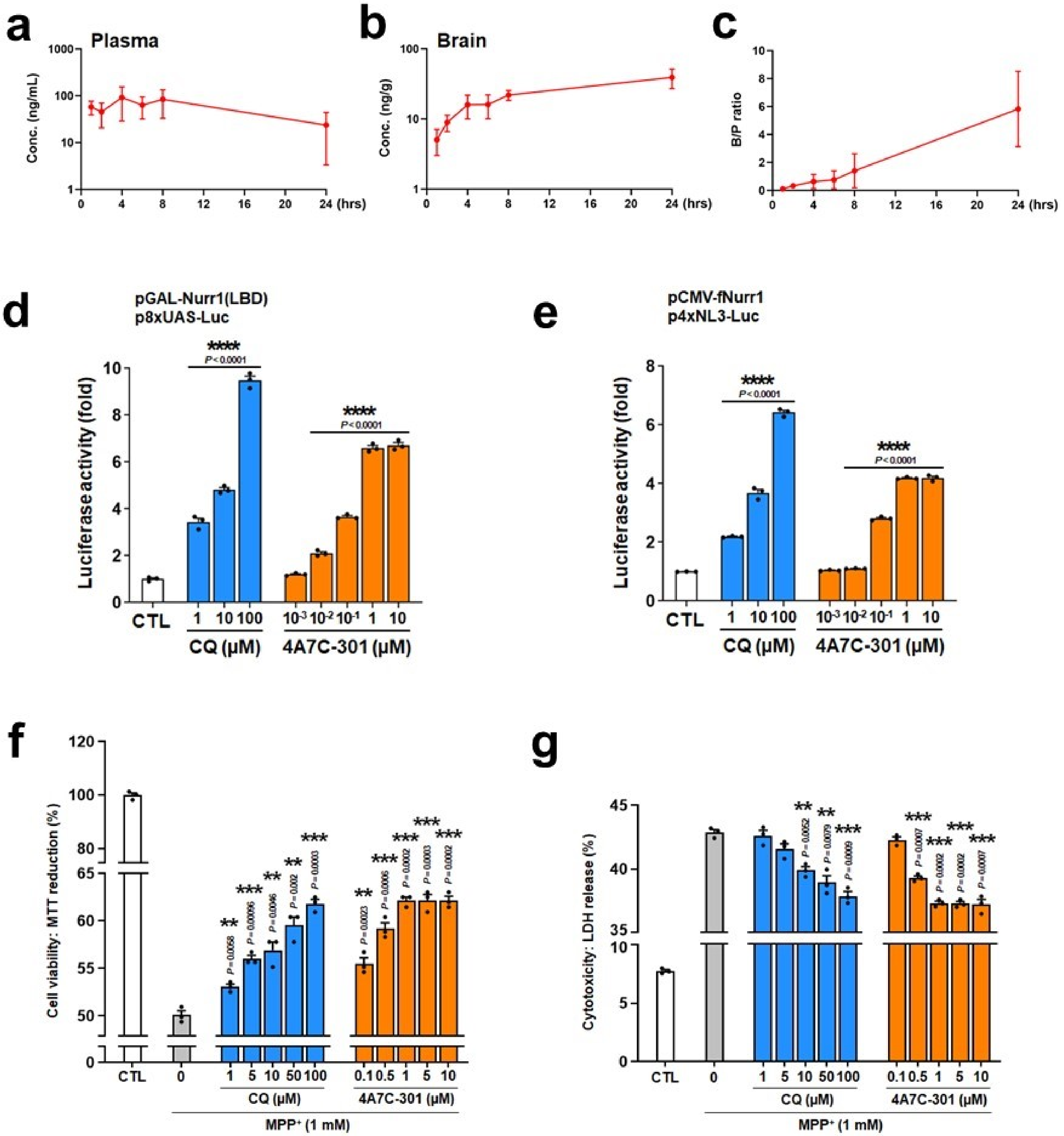

a
Plasma
Conc. (ng/mL)
24 (hrs)
b
Brain
Conc. (ng/g)
24 (hrs)
c
B/P ratio
24 (hrs)
d
pGAL-Nurr1(LBD)
p8xUAS-Luc
Luciferase activity (fold)
****
P < 0.0001
CTL
CQ (μM)
4A7C-301 (μM)
e
pCMV-fNurr1
p4xNL3-Luc
Luciferase activity (fold)
****
P < 0.0001
CTL
CQ (μM)
4A7C-301 (μM)
f
Cell viability: MTT reduction (%)
CTL
CQ (μM)
4A7C-301 (μM)
MPP+ (1 mM)
g
Cytotoxicity: LDH release (%)
CTL
CQ (μM)
4A7C-301 (μM)
MPP+ (1 mM)

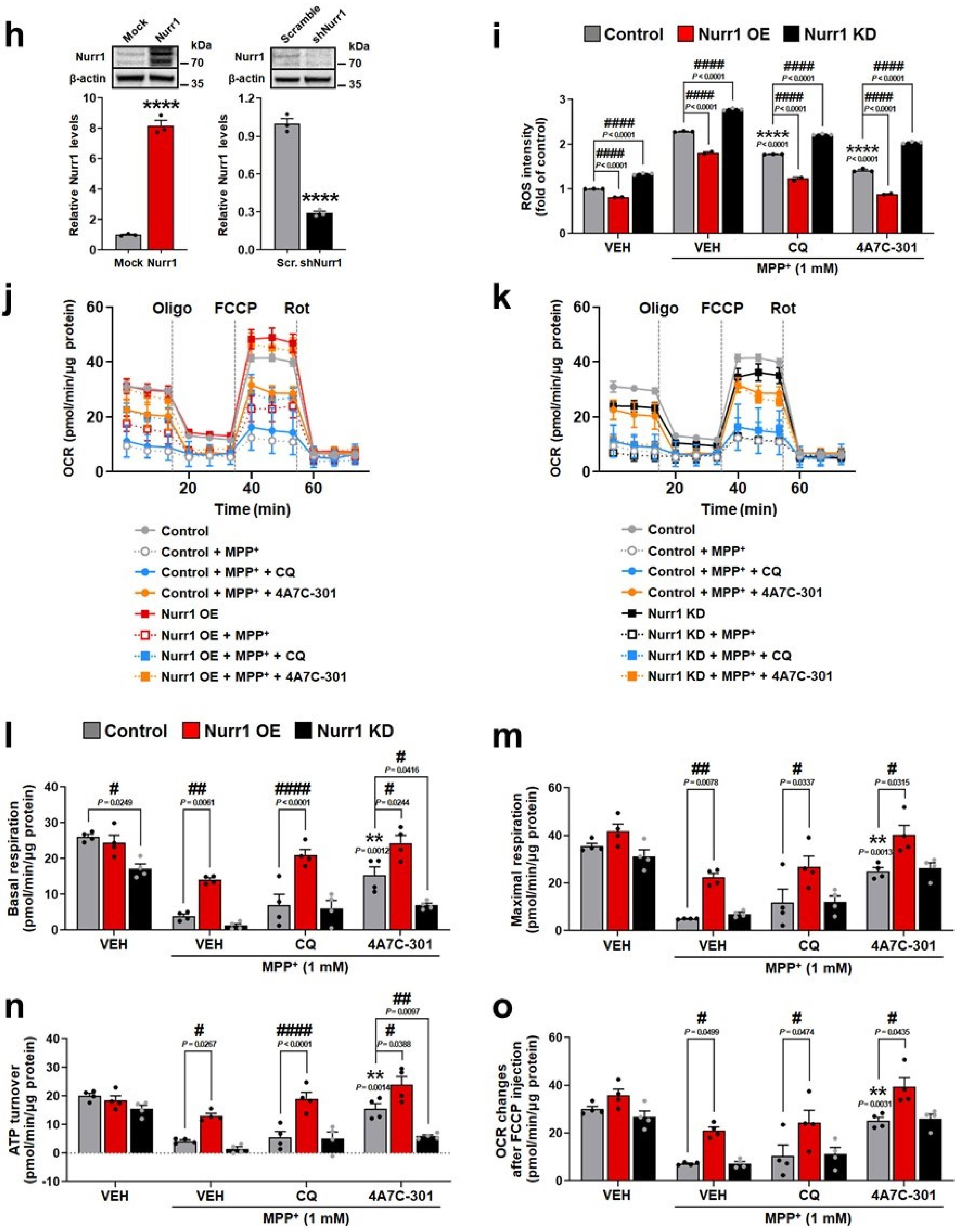
h
Mock Nurr1
Nurr1
β-actin
kDa
70
35
Relative Nurr1 levels
****
Mock Nurr1
Scramble shNurr1
Scr. shNurr1
i
Control Nurr1 OE Nurr1 KD
ROS intensity (fold of control)
P < 0.0001
****
VEH
VEH CQ 4A7C-301
MPP+ (1 mM)
j
k
Oligo FCCP Rot
OCR (pmol/min/μg protein)
Time (min)
Control
Control + MPP+
Control + MPP+ + CQ
Control + MPP+ + 4A7C-301
Nurr1 OE
Nurr1 OE + MPP+
Nurr1 OE + MPP+ + CQ
Nurr1 OE + MPP+ + 4A7C-301
Nurr1 KD
Nurr1 KD + MPP+
Nurr1 KD + MPP+ + CQ
Nurr1 KD + MPP+ + 4A7C-301
l
Control Nurr1 OE Nurr1 KD
Basal respiration (pmol/min/μg protein)
# P = 0.0249
P = 0.0061
P < 0.0001
# P = 0.0416
# P = 0.0244
** P = 0.0012
m
Maximal respiration (pmol/min/μg protein)
P = 0.0078
# P = 0.0337
# P = 0.0315
** P = 0.0013
n
ATP turnover (pmol/min/μg protein)
# P = 0.0267
P < 0.0001
P = 0.0097
# P = 0.0388
** P = 0.0014
o
OCR changes after FCCP injection (pmol/min/μg protein)
# P = 0.0499
# P = 0.0474
# P = 0.0435
** P = 0.0031
VEH
VEH CQ 4A7C-301
MPP+ (1 mM)

Supplementary Fig. 1. 4A7C-301 is brain-penetrant and potently activates Nurr1's transcriptional and neuroprotective function.

a-c, Blood-brain barrier (BBB) penetration analysis in SD rats (n = 3) determined by LC-MS/MS (liquid chromatography-tandem mass spectrometry) in the plasma (**a**) and the brain (**b**). The brain/plasma ratio (B/P ratio) (**c**) was calculated at each time point. Data are mean ± s.e.m.

d,e, Luciferase assays using Nurr1-LBD (**d**) or full-length Nurr1 (**e**) in N27-A cells. $^{***}P < 0.001$ compared to CTL, two-way ANOVA, Dunnett's *post-hoc* test; $n = 3$ biologically independent samples per group. Data are mean ± s.e.m.

f,g, Cell viability and cytotoxicity measured by MTT reduction assay (**f**) and LDH release assay (**g**), respectively in N27-A cells. $^{**}P < 0.01$, $^{***}P < 0.001$ compared to 0 μM, two-tailed unpaired *t*-test; $n = 3$ biologically independent samples per group. Data are mean ± s.e.m.

h, Nurr1 expression levels in Nurr1 OE or KD N27-A cells. $^{****}P < 0.0001$, Two-tailed Student's *t*-test; $n = 3$ biologically independent samples per group. Data are mean ± s.e.m.

i, ROS intensity detection by DCFDA staining in N27-A cells with Nurr1 OE or KD. Fluorescence intensity was normalized to control (Control VEH without MPP^+). $^{****}P < 0.0001$ compared to VEH treatment under Control conditions; $^{\#\#\#\#}P < 0.0001$, two-way ANOVA, Tukey's *post-hoc* test; $n = 3$ biologically independent samples per group. Data are mean ± s.e.m.

j,k, OXPHOS capacity measured using the Seahorse XFp analyzer in N27-A cells with Nurr1 OE (**j**) or KD (**k**). $n = 4$ biologically independent samples per group. Data are mean ± s.e.m.

l-o, Basal respiration (**l**), maximal respiration (**m**), ATP turnover (**n**), and OCR changes after FCCP injection (**o**) from N27-A cells with Nurr1 OE or KD shown in **j**,**k**. $^{**}P < 0.01$ compared to VEH treatment under Control condition; $^{\#}P < 0.05$, $^{\#\#}P < 0.01$, $^{\#\#\#\#}P < 0.0001$ compared between each treatment group, two-way ANOVA, Tukey's *post-hoc* test; $n = 4$ biologically independent samples per group. Data are mean ± s.e.m.

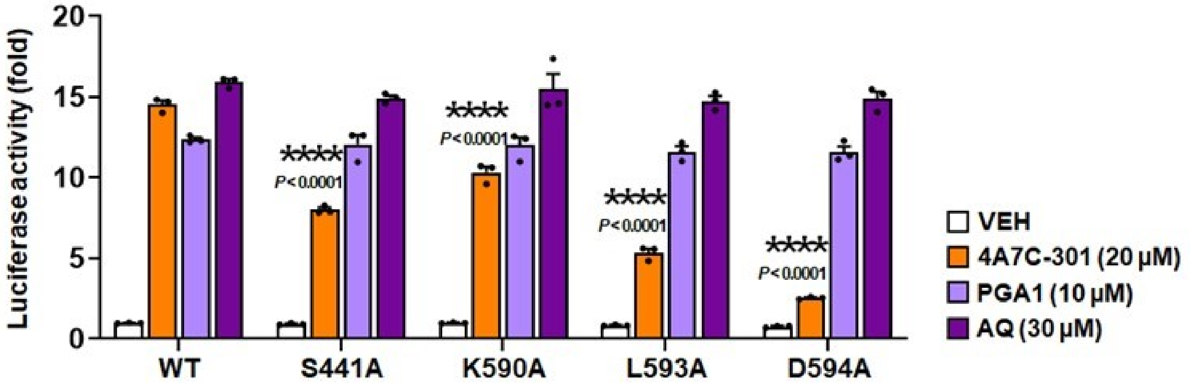

Supplementary Fig. 2. Effect of 4A7C-301 and other Nurr1 agonists on the transcriptional activities of mutant Nurr1-LBD constructs in SK-N-BE(2)C cells.
***$P < 0.001$ compared to wild-type (WT). Two-way ANOVA, Dunnett's *post-hoc* test; $n = 3$ biologically independent samples per group. Data are mean ± s.e.m.

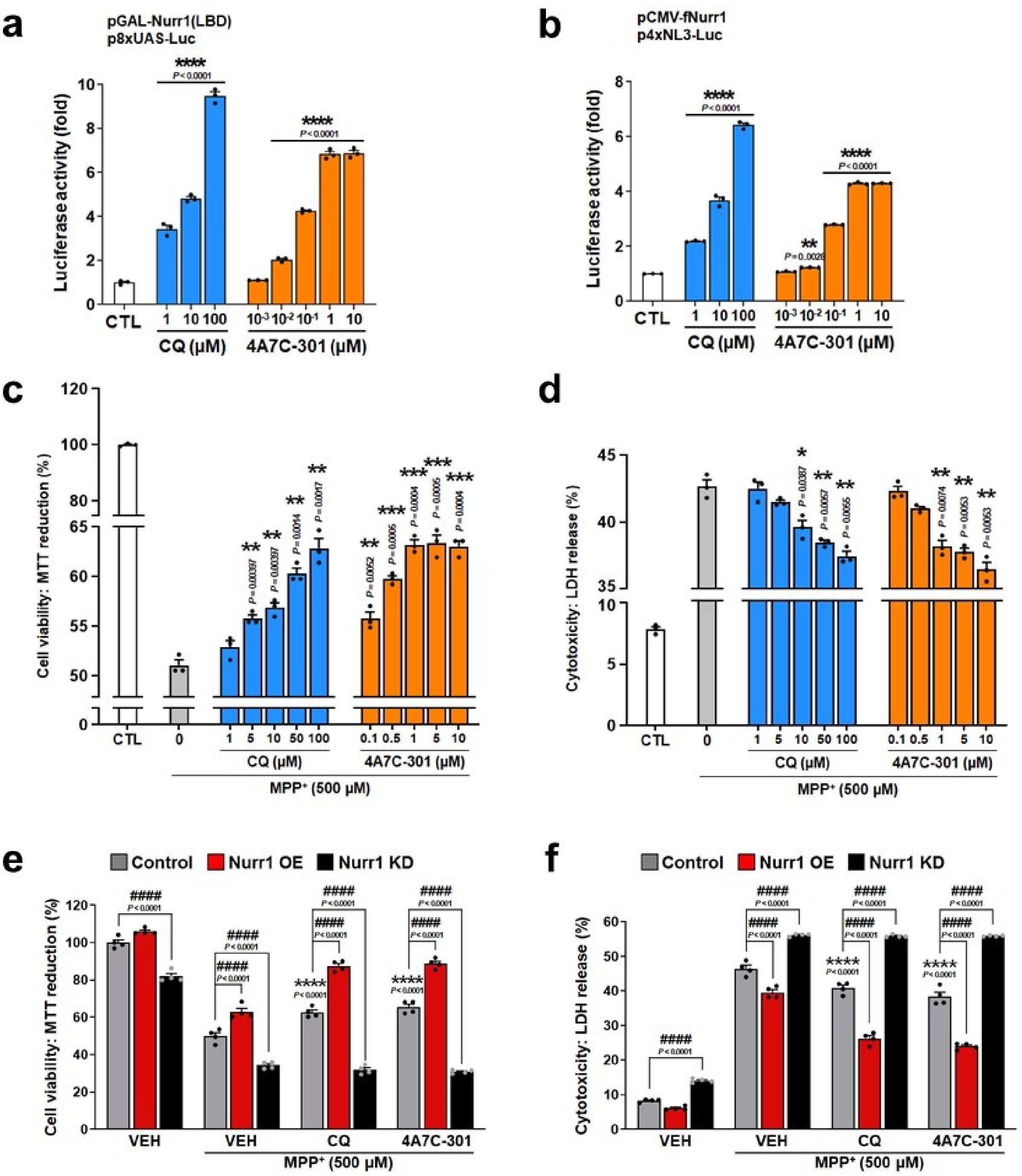

Supplementary Fig. 3. 4A7C-301 exhibits highly potent transactivation and neuroprotection compared to CQ.

a,b, Luciferase assays using Nurr1-LBD (**a**) or full-length Nurr1 (**b**) in MN9D cells. $^{**}P < 0.01$, $^{****}P < 0.0001$ compared to control (CTL), two-way ANOVA, Dunnett's *post-hoc* test; $n = 3$ biologically independent samples per group. Data are mean ± s.e.m.

c,d, Cell viability and cytotoxicity measured by MTT reduction assay (**c**) and LDH release assay (**d**), respectively in MN9D cells. $^{*}P < 0.05$, $^{**}P < 0.01$, $^{***}P < 0.001$ compared to 0 μM, two-tailed unpaired *t*-test; $n = 3$ biologically independent samples per group. Data are mean ± s.e.m.

e,f, Cell viability analyzed by MTT reduction (**e**) and cytotoxicity measured using LDH release (**f**) with Nurr1 OE and KD in MN9D cells. $^{****}P < 0.001$ compared to vehicle (VEH) treatment under Control conditions; $^{\#\#\#\#}P < 0.0001$, two-way ANOVA, Tukey's *post-hoc* test; $n = 4$ biologically independent samples per group. Data are mean ± s.e.m.

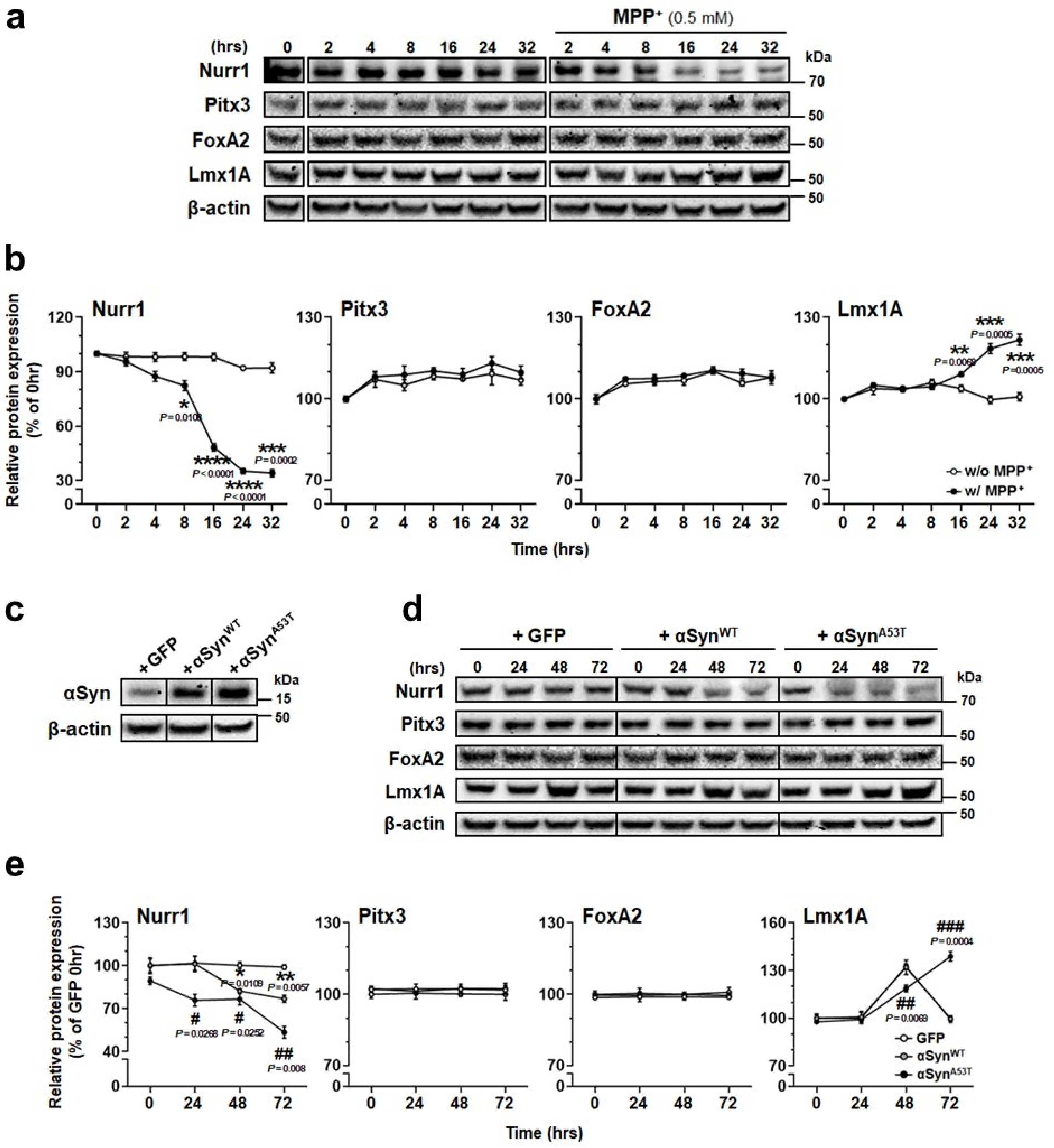
a
MPP+ (0.5 mM)
(hrs) 0 2 4 8 16 24 32 2 4 8 16 24 32
Nurr1
Pitx3
FoxA2
Lmx1A
β-actin
kDa
70
50
b
Nurr1
Pitx3
FoxA2
Lmx1A
Relative protein expression (% of 0hr)
P=0.0108
P<0.0001
P=0.0002
P=0.0005
w/o MPP+
w/ MPP+
Time (hrs)
c
+GFP
+αSynWT
+αSynA53T
αSyn
β-actin
kDa
15
50
d
+ GFP
+ αSynWT
+ αSynA53T
(hrs) 0 24 48 72 0 24 48 72 0 24 48 72
Nurr1
Pitx3
FoxA2
Lmx1A
β-actin
kDa
70
50
e
Nurr1
Pitx3
FoxA2
Lmx1A
Relative protein expression (% of GFP 0hr)
P=0.0109
P=0.0057
P=0.0268
P=0.0252
P=0.008
P=0.0004
P=0.0069
GFP
αSynWT
αSynA53T
Time (hrs)

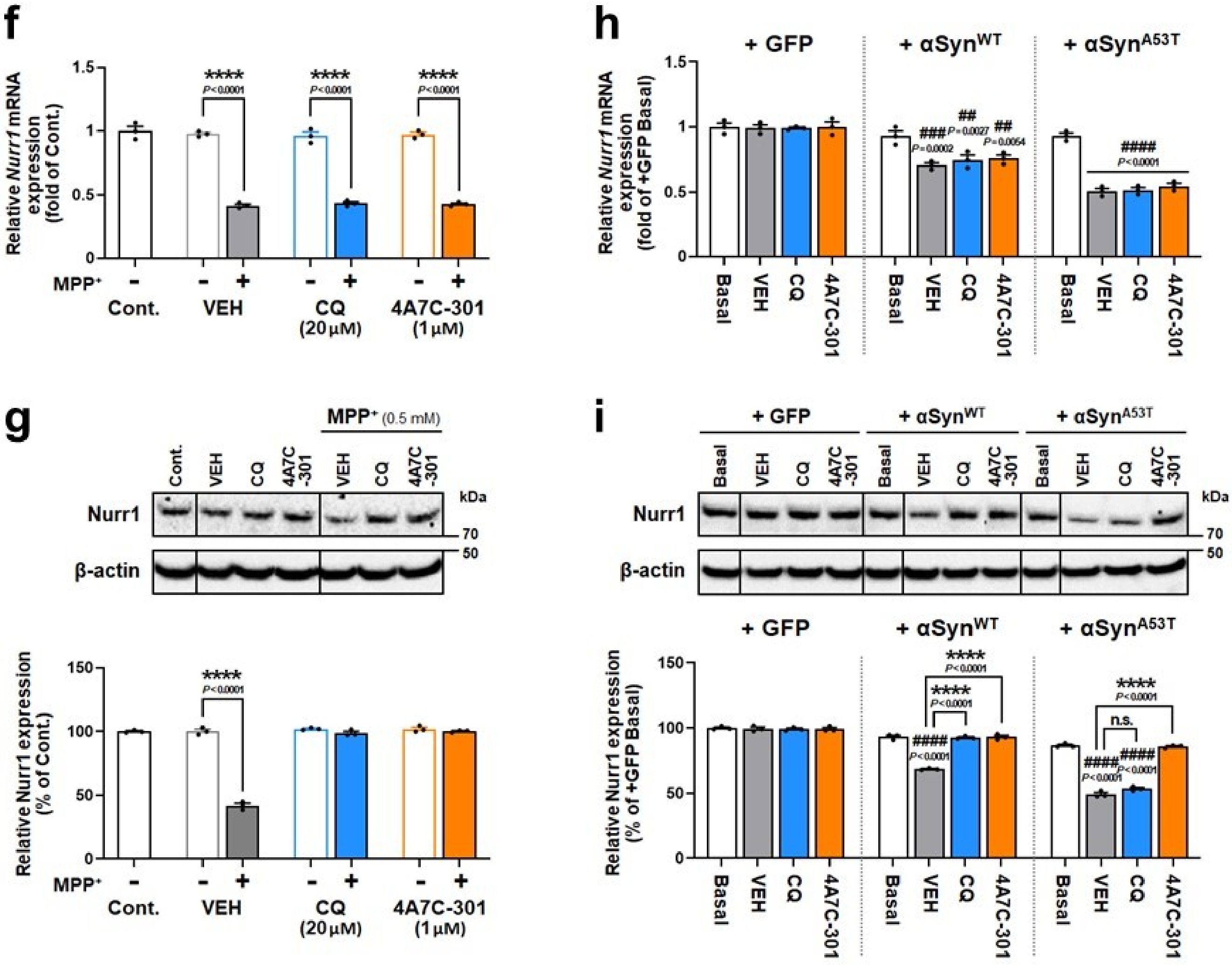

Supplementary Fig. 4. Environmental (MPP$^+$) and genetic (αSyn) risk factors of PD compromise the level of Nurr1 but not those of other key transcription factors in MN9D cells.

a,b, Western blot analyses of expression level changes of key mDANs transcription factors against prolonged exposure to MPP$^+$. **P* < 0.05, ***P* < 0.01, ****P* < 0.001, *****P* < 0.0001 compared to w/o MPP$^+$, two-way ANOVA, Sidak's multiple comparisons; *n* = 3 biologically independent samples per group. Data are mean ± s.e.m.

c-e, Western blot analyses of expression level changes of key mDANs transcription factors against overexpression of WT or mutant αSyn. **P* < 0.05, ***P* < 0.01 compared between GFP and WT αSyn (αSyn^{WT}); $^{\#\#}P < 0.05$, $^{\#\#}P < 0.01$, $^{\#\#\#}P < 0.001$ compared between GFP and mutant αSyn (αSyn^{A53T}), two-way ANOVA, Sidak's multiple comparisons; *n* = 3 biologically independent samples per group. Data are mean ± s.e.m.

f,g, Real-time PCR (**f**) and Western blot (**g**) analyses of Nurr1 expression changes after CQ or 4A7C-301 treatment in the absence or presence of MPP$^+$. *****P* < 0.0001, one-way ANOVA, Tukey's multiple comparisons; *n* = 3 biologically independent samples per group. Data are mean ± s.e.m.

h,i, Real-time PCR (**h**) and Western blot (**i**) analyses of Nurr1 expression changes after CQ or 4A7C-301 treatment under WT or mutant αSyn overexpression. $^{\#\#}P < 0.01$, $^{\#\#\#}P < 0.001$, $^{\#\#\#\#}P < 0.0001$ compared to Basal; *****P* < 0.0001; *n.s.*, not significant (*P* > 0.05), two-way ANOVA, Tukey's multiple comparisons; *n* = 3 biologically independent samples per group. Data are mean ± s.e.m.

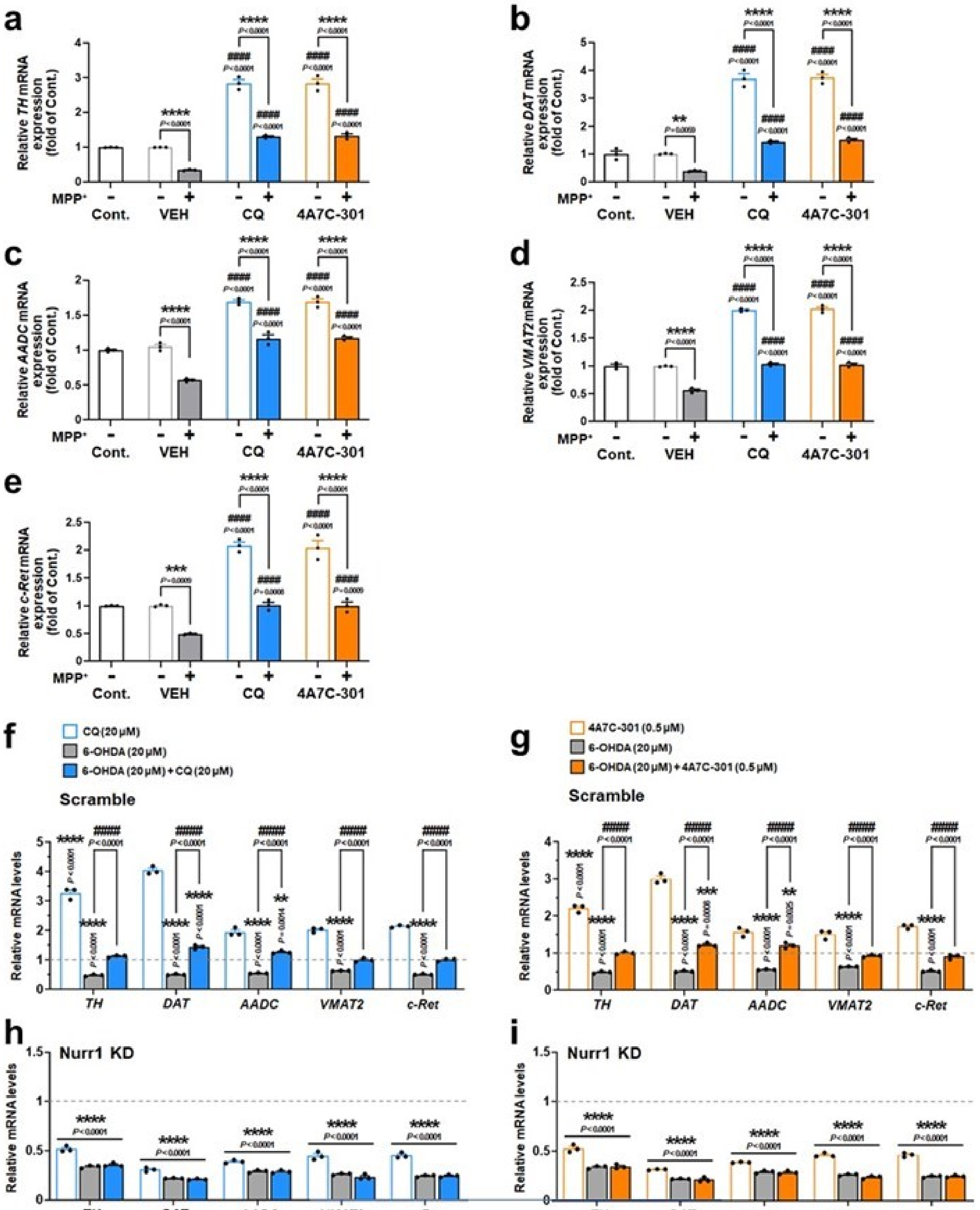

Supplementary Fig. 5. Effects of CQ and 4A3C-301 on dopaminergic gene expression in MN9D cells and mouse ventral mesencephalic (VM) primary cells.

a-e, Real-time PCR analyses of tyrosine hydroxylase (*TH*) (**a**), dopamine transporter (*DAT*) (**b**), aromatic L-amino acid decarboxylase (*AADC*) (**c**), vesicular monoamine transporter 2 (*VMAT2*) (**d**) and *c-Ret* (**e**) expression after CQ or 4A7C-301 treatment in the absence or presence of MPP^+ in MN9D cells. $**P < 0.01$, $***P < 0.001$, $****P < 0.0001$; $^{####}P < 0.0001$ compared to VEH. One-way ANOVA, Dunnett's multiple comparisons; $n = 3$ biologically independent samples per group. Data are mean ± s.e.m.

f-i, Real-time PCR analyses of *TH*, *DAT*, *AADC*, *VMAT2* and *c-Ret* expression after CQ or **4A7C-301** treatment in the absence or presence of 6-OHDA under normal (**f,g**) or Nurr1 knocked down (**h,i**) conditions. in VM primary cells. $^{####}P < 0.0001$; $**P < 0.01$, $****P < 0.0001$ compared to VEH treatment in Scramble condition. Two-way ANOVA, Dunnett's *post-hoc* test; $n = 3$ biologically independent samples per group. Data are mean ± s.e.m.

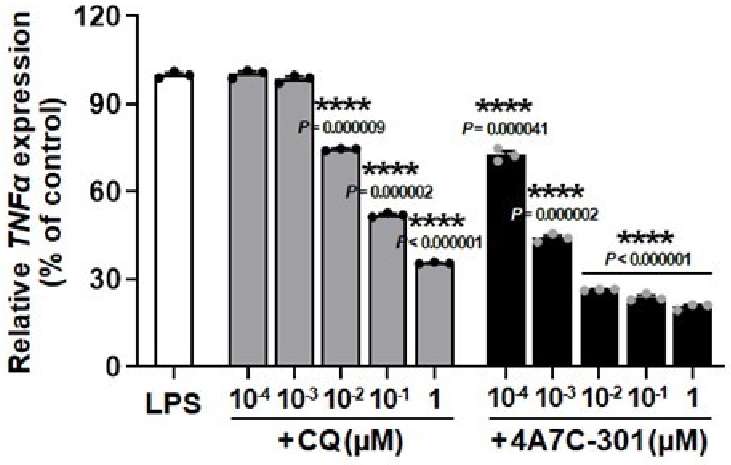

Supplementary Fig. 6. Comparison of CQ and 4A7C-301 for their suppressive function of LPS-induced *TNFα* gene expression in BV2 cells.

The Ct values to calculate the relative value of LPS-treated control group are 20.76 for *GAPDH* and 24.85 for *TNFα*.****$P < 0.0001$ compared to LPS only, two-tailed unpaired t-test; $n = 3$ biologically independent samples per group. Data are mean ± s.e.m.

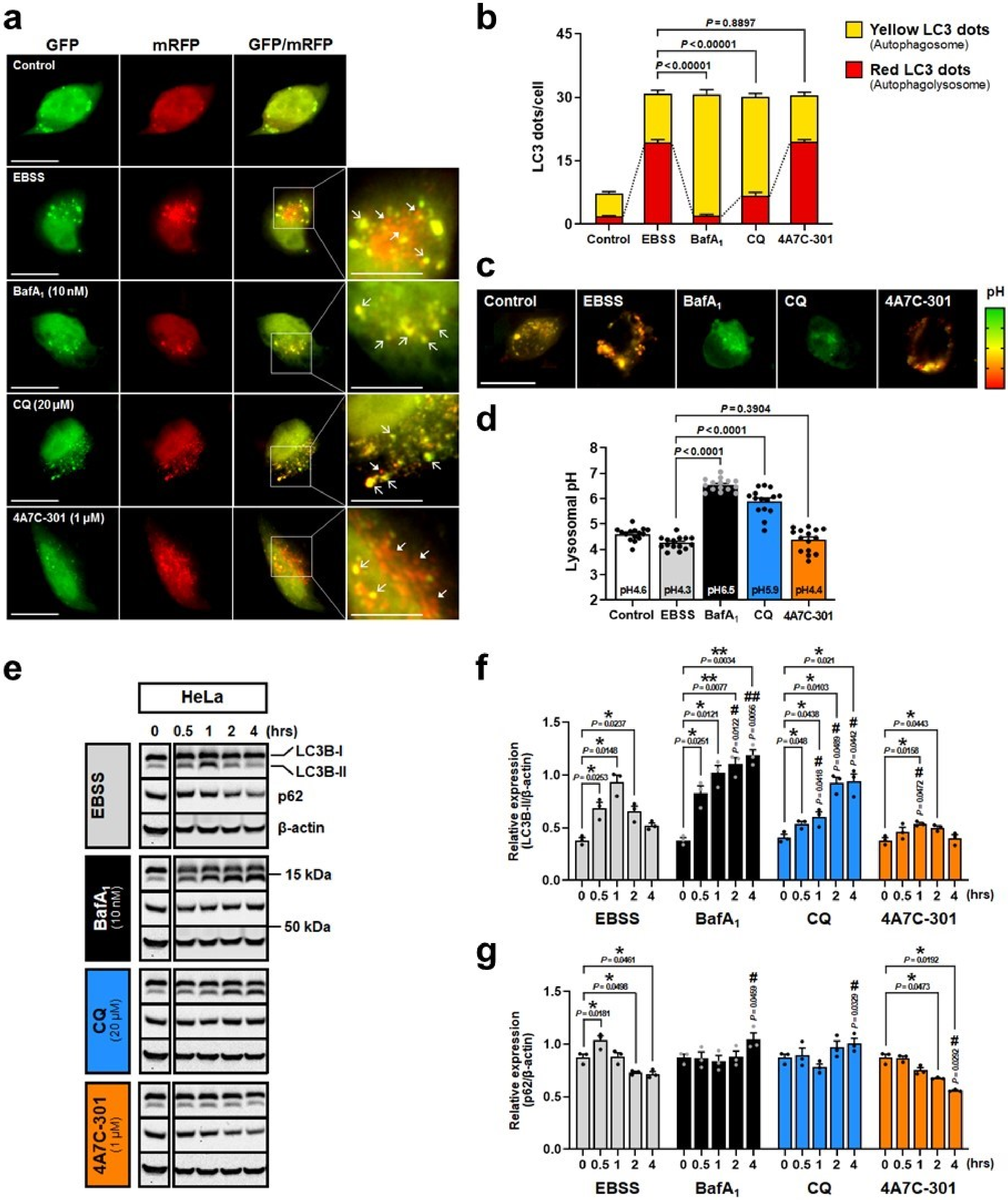
a
GFP
mRFP
GFP/mRFP
Control
EBSS
BafA1 (10 nM)
CQ (20 μM)
4A7C-301 (1 μM)
b
Yellow LC3 dots
(Autophagosome)
Red LC3 dots
(Autophagolysosome)
LC3 dots/cell
P = 0.8897
P < 0.00001
P < 0.00001
Control
EBSS
BafA1
CQ
4A7C-301
c
Control
EBSS
BafA1
CQ
4A7C-301
pH
d
Lysosomal pH
P = 0.3904
P < 0.0001
P < 0.0001
pH4.6
pH4.3
pH6.5
pH5.9
pH4.4
Control
EBSS
BafA1
CQ
4A7C-301
e
HeLa
0 0.5 1 2 4 (hrs)
LC3B-I
LC3B-II
p62
β-actin
15 kDa
50 kDa
EBSS
BafA1 (10 nM)
CQ (20 μM)
4A7C-301 (1 μM)
f
Relative expression (LC3B-II/β-actin)
0 0.5 1 2 4 (hrs)
EBSS
BafA1
CQ
4A7C-301
g
Relative expression (p62/β-actin)
0 0.5 1 2 4 (hrs)
EBSS
BafA1
CQ
4A7C-301

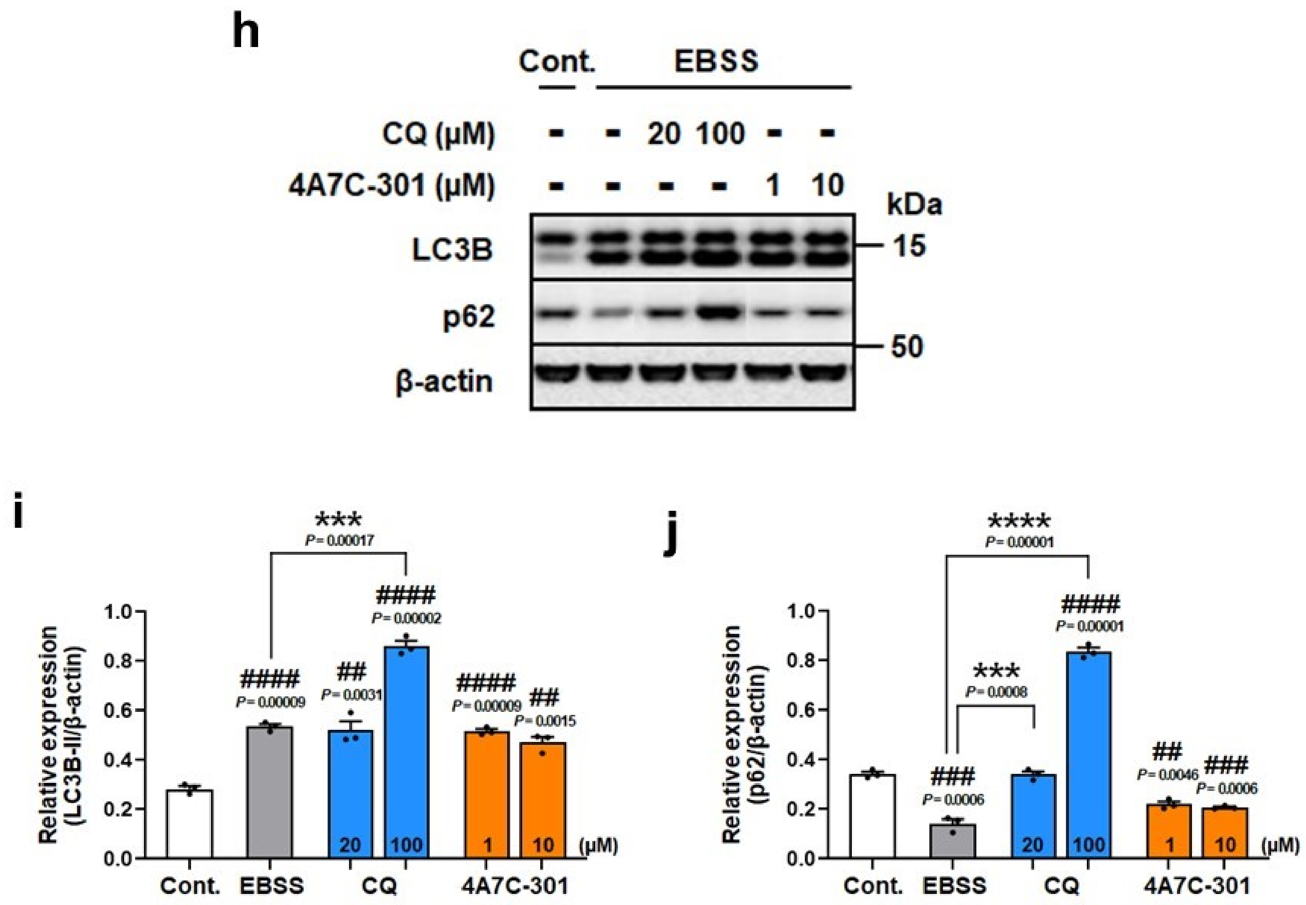

Supplementary Fig. 7. Altered autophagy process by CQ but not by 4A7C-301 in HeLa cells.
a,b, Autophagolysosome (APL) formation assay. **a,** Tandem mRFP-GFP-LC3 fluorescence images for APL detection. Scale bars, 20 µm. **b,** Number of yellow LC3 dots and red LC3 dots per cell was counted from 10 random cells in each well from triplicates for each condition (total of 30 cells per each group). Two-tailed unpaired *t*-test. Data are mean ± s.e.m.
c,d, Lysosomal pH detection. **c,** LysoSensor™ Yellow/Blue DND-160 fluorescence images. Scale bars, 20 µm. **d**, Quantification from 5 random cells in each well from triplicates for each treatment group (total of 15 cells per each group). Two-tailed unpaired *t*-test. Data are mean ± s.e.m.
e-g, Autophagic flux analyses. Autophagic flux markers LC3B and p62 (**e**) and its expression levels were quantified (**f,g**). $*P < 0.05$, $**P < 0.01$; $^{\#}P < 0.05$, $^{\#\#}P < 0.01$ compared to EBSS, two-way ANOVA, Dunnett's multiple comparisons; $n = 3$ biologically independent samples per group. Data are mean ± s.e.m.
h-j, Effects of different concentrations of CQ and 4A7C-301 on autophagic flux in HeLa cells determined by Western blot (**h**) and quantification of LC3B-II (**i**) and p62 (**j**). $^{\#\#}P < 0.01$, $^{\#\#\#}P < 0.001$, $^{\#\#\#\#}P < 0.0001$ compared to control (Cont.); $***P < 0.001$, $****P < 0.0001$ compared to EBSS, two-tailed unpaired *t*-test; $n = 3$ biologically independent samples per group. Data are mean ± s.e.m.

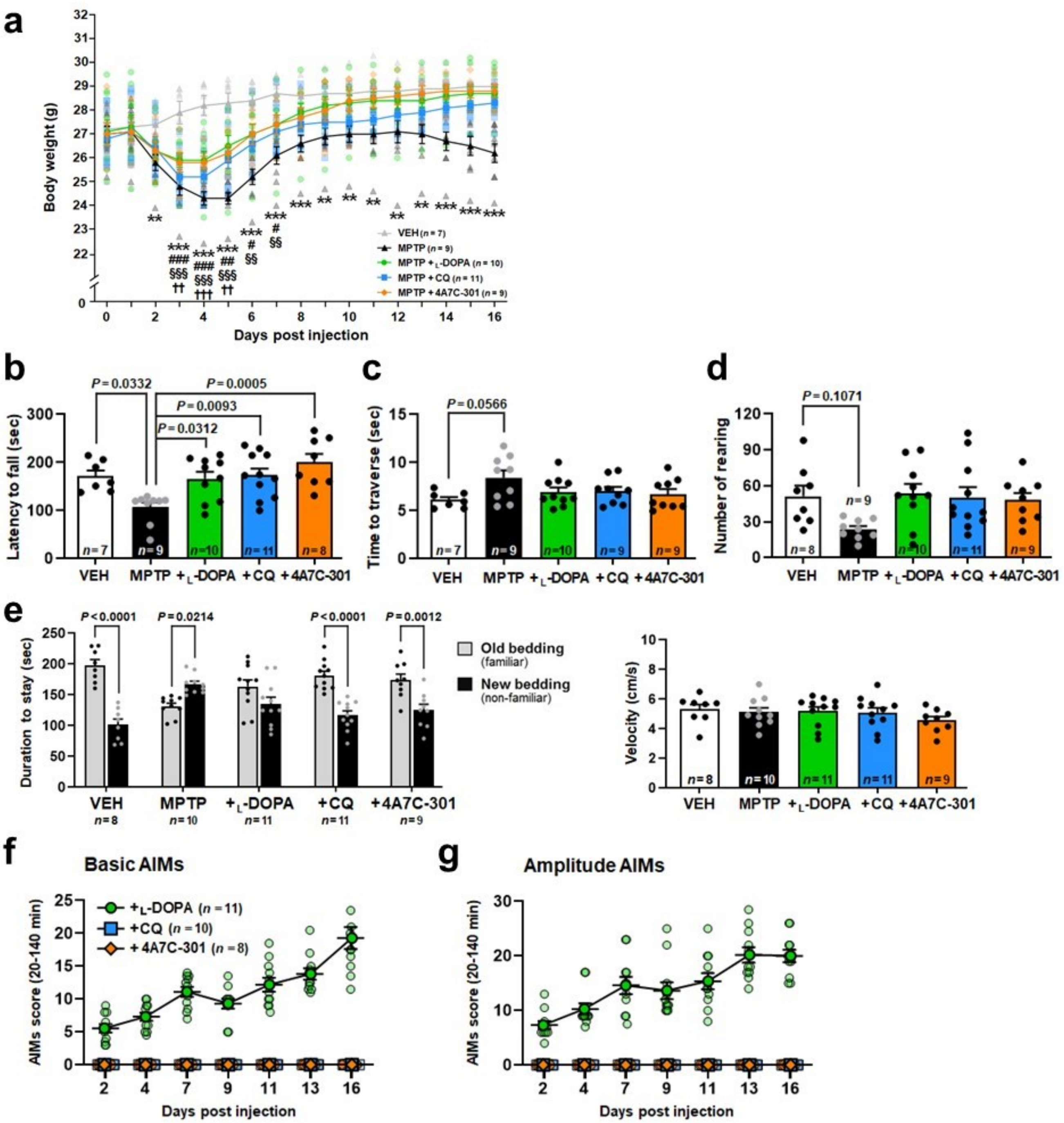

Supplementary Fig. 8. Assessment of motor and non-motor behaviors at the chronic stage (D14-D15) of MPTP-induced mice.

a, Body weight changes of MPTP-induced PD model mice treated with vehicle (VEH), L-DOPA, CQ or 4A7C-301. $^{**}P < 0.01$, $^{***}P < 0.001$ compared between VEH and MPTP; $^{\#}P < 0.05$, $^{\#\#}P < 0.01$, $^{\#\#\#}P < 0.001$ compared between VEH and MPTP + L-DOPA; $^{\S\S}P < 0.01$, $^{\S\S\S}P < 0.001$ compared between VEH and MPTP + CQ; $^{\dagger\dagger}P < 0.01$, $^{\dagger\dagger\dagger}P < 0.001$ compared between VEH and MPTP + 4A7C-301. Two-way ANOVA, Sidak's *post-hoc* test. Data are mean ± s.e.m.

b-d, Motor deficits-related behavior tests including rotarod (**b**), pole test (**c**), and cylinder test (**d**) at the chronic stage (D15). $^{*}P < 0.05$ compared to VEH; One-way ANOVA, Tukey's *post-hoc* test; $n \geq 7$ per group. Data are mean ± s.e.m.

e, Olfactory discrimination (left) and velocity (right) at the chronic stage (D14). One-way ANOVA, Tukey's *post-hoc* test; $n \geq 7$ per group. Data are mean ± s.e.m.

f,g, Basic (**f**) and amplitude (**g**) AIMs scores. $n \geq 7$ per group. Data are mean ± s.e.m.

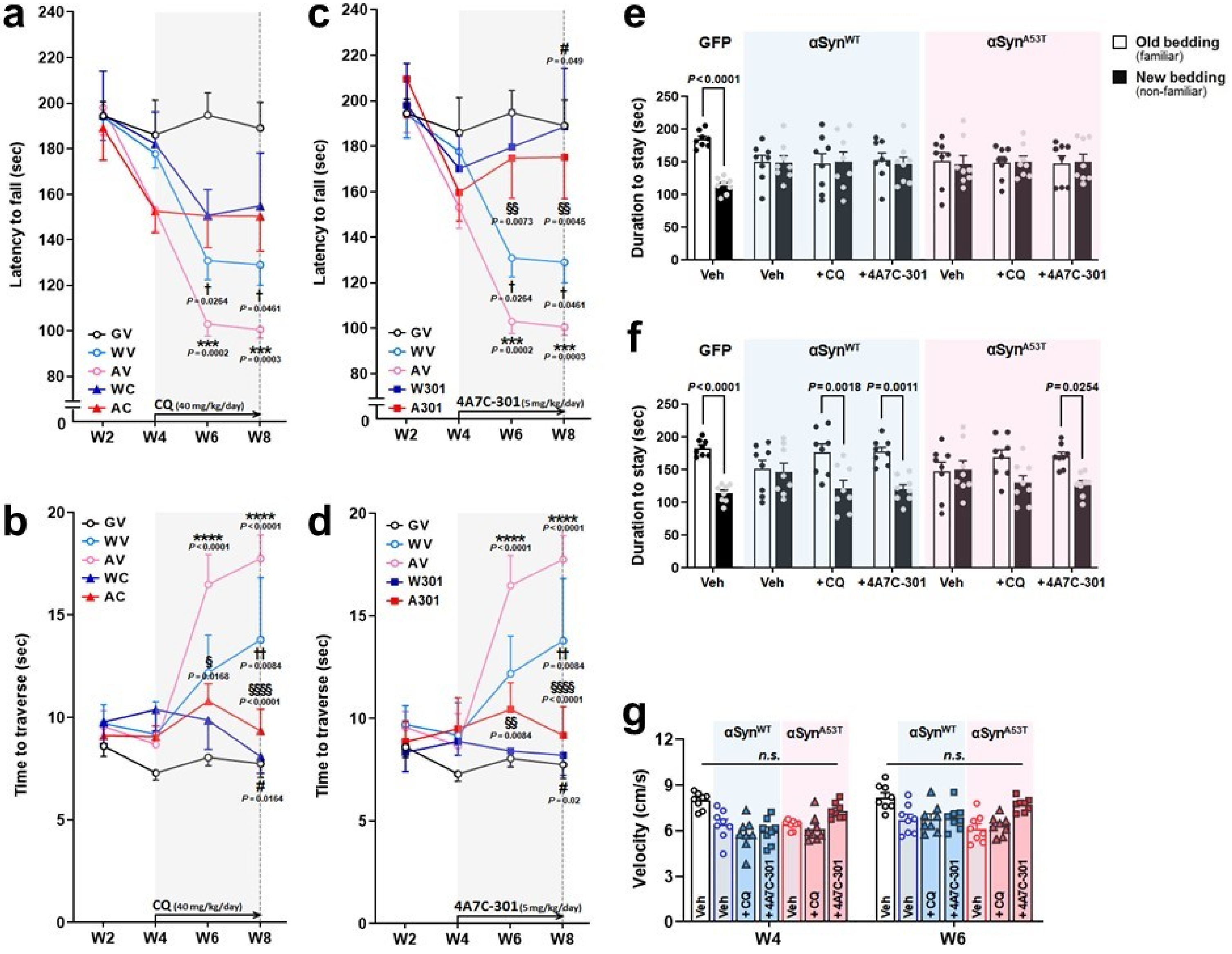
a
b
c
d
e
f
g
Latency to fall (sec)
Time to traverse (sec)
Duration to stay (sec)
Velocity (cm/s)
GV
WV
AV
WC
AC
W301
A301
W2
W4
W6
W8
CQ (40 mg/kg/day)
4A7C-301 (5 mg/kg/day)
GFP
αSynWT
αSynA53T
Old bedding (familiar)
New bedding (non-familiar)
Veh
+CQ
+4A7C-301
P<0.0001
P=0.0018
P=0.0011
P=0.0254
n.s.
W4
W6

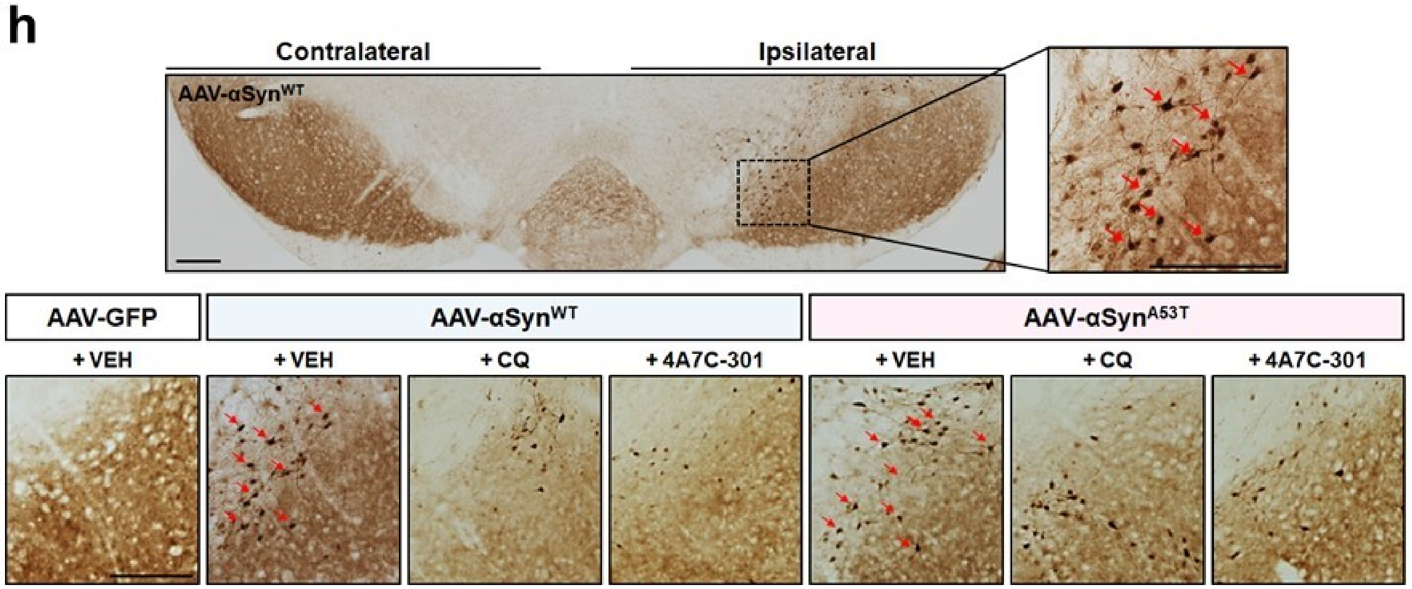

Supplementary Fig. 9. Recovery of motor and non-motor deficits by CQ and 4A7C-301 in AAV-αSyn-induced PD model mice.
a-d, Rotarod test (**a,c**) and pole test (**b,d**) with CQ (**a,b**) or 4A7C-301 (**c,d**) treated group. $^{\dagger}P < 0.05$, $^{\dagger\dagger}P < 0.01$ compared between GV and WV; $^{***}P < 0.001$, $^{****}P < 0.0001$ compared between GV and AV; $^{\#}P < 0.05$ compared WV to WC or W301; $^{\S\S}P < 0.01$, $^{\S\S\S\S}P < 0.0001$ compared AV to AC or A301. Two-way ANOVA, Tukey's multiple comparisons; $n = 8$ per group. Data are mean ± s.e.m.
e,f, Olfactory discrimination tests performed at 4 weeks (**e**) and 6 weeks (**f**) after surgery. Evident olfactory disfunction by αSyn^{WT} or αSyn^{A53T} expression is observed from 4 weeks after surgery. Two-way ANOVA, Tukey's multiple comparisons; $n = 8$ per group. Data are mean ± s.e.m. GV, GFP + VEH; WV, αSyn^{WT} + VEH; AV, αSyn^{A53T} + VEH; WC, αSyn^{WT} + CQ; AC, αSyn^{A53T} + CQ; W301, αSyn^{WT} + 4A7C-301; A301, αSyn^{A53T} + 4A7C-301.
g, Velocities during the session at 4 weeks and 6 weeks. *n.s.*, not significant ($P > 0.05$), one-way ANOVA; $n = 8$ per group. Data are mean ± s.e.m.
h, Representative pS129 immunohistochemistry from two independent experiments in the SNpc. Arrows indicate $pS129^{+}$ cells. Bottom panel is presented in Fig. 5l. Scale bars, 250 μm.

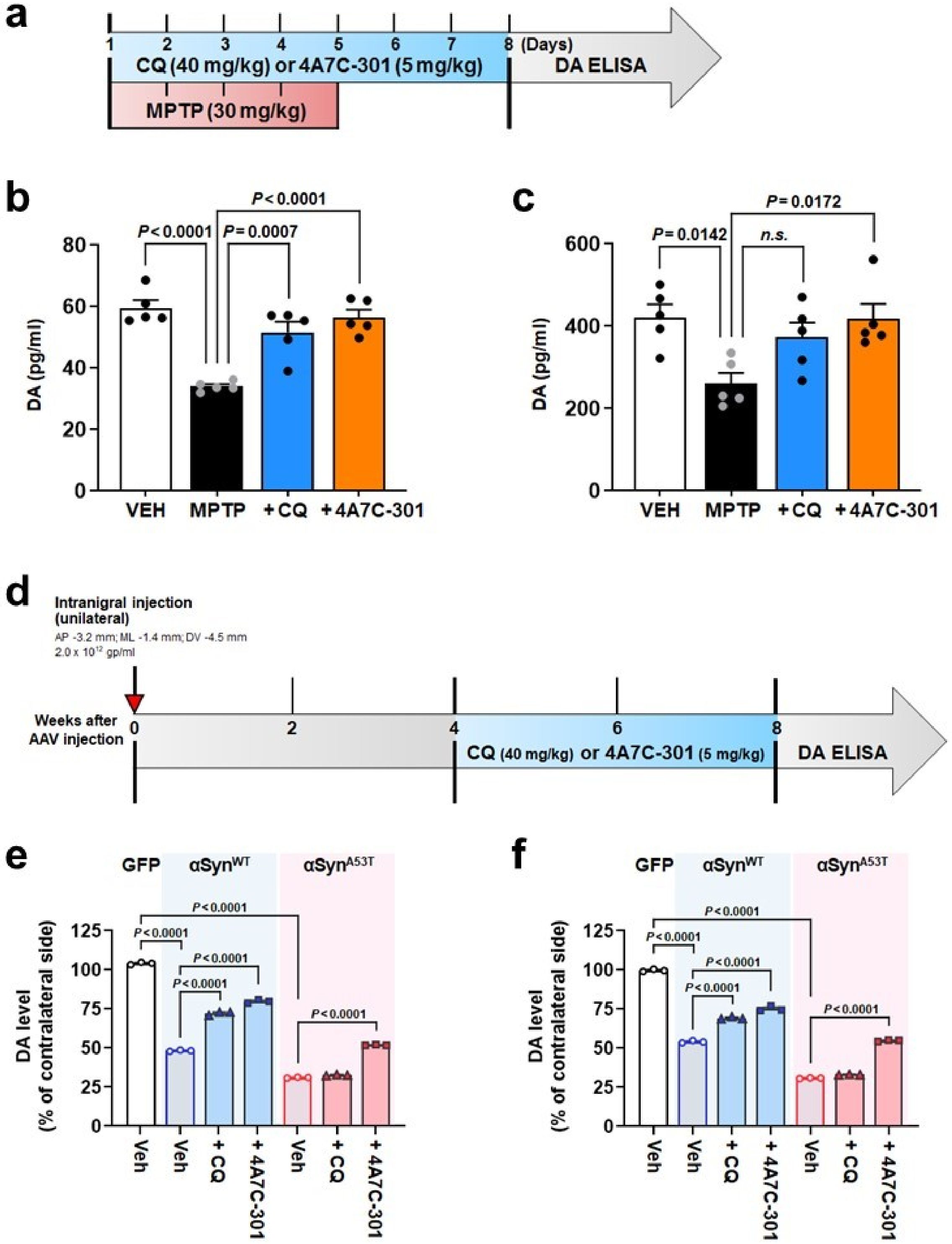

Supplementary Fig. 10. Restoration of reduced DA levels by CQ and 4A7C-301 in MPTP- (a-c) and αSyn-induced (d-f) mouse models.

a, Schematic representation of CQ and 4A7C-301 administrations to MPTP-treated mice.

b,c, DA ELISA in the SN (**b**) and STR (**c**). *n.s.*, not significant ($P > 0.05$). One-way ANOVA, Tukey's *post-hoc* test; $n = 5$ per group. Data are mean ± s.e.m.

d, Schematic representation of CQ and 4A7C-301 administrations to AAV-αSyn-injected mice.

e,f, DA ELISA in the SN (**e**) and STR (**f**). One-way ANOVA, Tukey's *post-hoc* test; $n = 3$ per group. Data are mean ± s.e.m.

Supplementary Table 1. Structure modification information, EC_{50} and maximal activity of top 36 compounds selected from > 570 4A7C-derivatives.

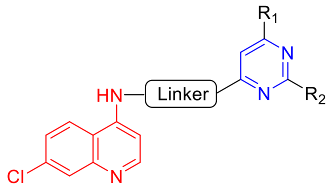

4A7C-Pyrimidine Regiomer-1

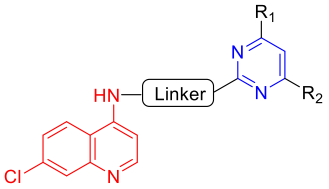

4A7C-Pyrimidine Regiomer-2

S. No.	ID	Pyrimidine regiochemistry	Linker	Pyrimidine substituent R_1	Pyrimidine substituent R_2	EC_{50}	Maximum fold activity
	CQ	-	-	-	-	50.25 ± 11-13 μM	16.84 ± 2.57
1	4A7C-101	Regiomer-1	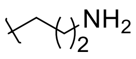	Me	Piperidine	121.22 ± 56.69 nM	5.08 ± 0.70
2	4A7C-102	Regiomer-1	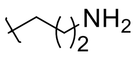	Me	N-Et piperazine	143.13 ± 2.88 nM	3.68 ± 0.04
3	4A7C-103	Regiomer-2	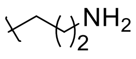	Cl	N-Ethyl piperazine	634.46 ± 1.37 nM	3.52 ± 0.19
4	4A7C-104	Regiomer-2	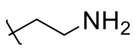	Cl	N-Ethyl piperazine	315.67 ± 39.76 nM	2.63 ± 0.58
5	4A7C-201	Regiomer-1	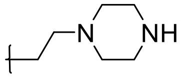	Me	N-Methyl piperazine	14.59 ± 1.38 nM	4.78 ± 0.31
6	4A7C-202	Regiomer-1	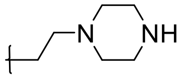	Me	Pyrrolidine	1.28 ± 0.63 nM	3.78 ± 0.25
7	4A7C-203	Regiomer-1	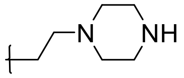	H	Cl	17.02 ± 4.97 nM	4.22 ± 0.16
8	4A7C-204	Regiomer-1	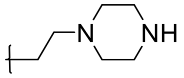	H	Thiomorpholine	33.69 ± 0.34 nM	4.46 ± 0.67
9	4A7C-205	Regiomer-1	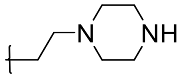	H	1H-Imidazole	1.32 ± 0.31 μM	4.64 ± 0.21
10	4A7C-206	Regiomer-1	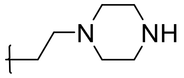	H	N-Ethyl piperazine	10.03 ± 0.76 μM	10.71 ± 0.44
11	4A7C-508	Regiomer-2	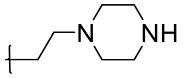	H	Thiomorpholine	1.46 ± 0.97 μM	5.50 ± 0.14
12	4A7C-509	Regiomer-2	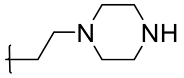	H	1H-Imidazole	112.57 ± 19.21 nM	3.43 ± 0.42
13	**4A7C-301**	Regiomer-2	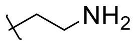	N-Et Piperazine	N-Et Piperazine	6.53 ± 0.33 μM	18.12 ± 3.02
14	4A7C-302	Regiomer-2	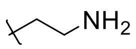	Piperidine	Piperidine	950.63 ± 6.03 nM	5.19 ± 0.18

15	4A7C-303	Regiomer-1	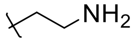	N-Et Piperazine	N-Et Piperazine	8.50 ± 18.62 nM	9.50 ± 0.22
16	4A7C-304	Regiomer-2	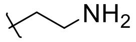	Piperidine	N-Et Piperazine	9.67 ± 7.87 nM	2.16 ± 0.03
17	4A7C-305	Regiomer-2	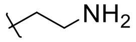	Morpholine	N-Et Piperazine	1.04 ± 4.19 μM	7.60 ± 0.08
18	4A7C-306	Regiomer-2	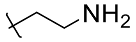	N-Me Piperazine	N-Et Piperazine	25.46 ± 42.21 nM	10.01 ± 0.06
19	4A7C-401	Quinazoline ring	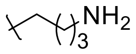	NA	N-Et Piperazine	2.62 ± 0.96 nM	3.01 ± 0.13
20	4A7C-501	Regiomer-1	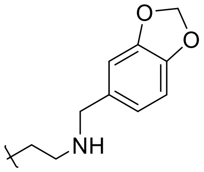	Me	N-Me Piperazine	3.39 ± 0.26 μM	10.51 ± 0.85
21	4A7C-502	Regiomer-1	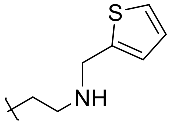	Me	Cl	0.51 ± 0.06 nM	4.99 ± 0.86
22	4A7C-503	Regiomer-2	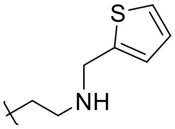	Me	Morpholine	1.07 ± 0.12 nM	2.25 ± 0.26
23	4A7C-504	Regiomer-2	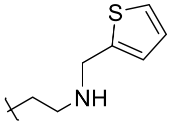	Me	N-Me Piperazine	431.29 ± 15.14 nM	4.07 ± 0.53
24	4A7C-505	Regiomer-1	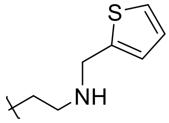	Me	Piperidine	2.14 ± 0.45 nM	3.65 ± 0.31
25	4A7C-506	Regiomer-1	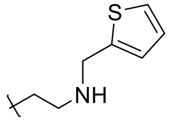	Me	Morpholine	9.83 ± 0.29 nM	4.49 ± 0.43
26	4A7C-507	Regiomer-2	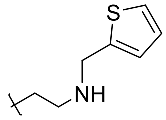	Me	N-Et Piperazine	94.87 ± 1.66 nM	3.24 ± 0.40
27	4A7C-508	Regiomer-2	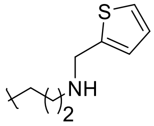	Me	Thiomorpholine	1.54 ± 0.42 μM	5.29 ± 0.06
28	4A7C-601	Regiomer-1	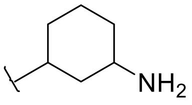	Me	Cl	109.82 ± 27.07 nM	3.81 ± 0.17

29	4A7C-602	Regiomer-1	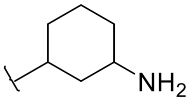	Me	N-Ph Piperazine	3.86 ± 0.26 μM	3.44 ± 0.28
30	4A7C-603	Regiomer-1	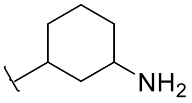	Me	Piperidine	1.22 ± 0.02 μM	6.84 ± 0.52
31	4A7C-604	Regiomer-2	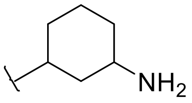	Me	Pyrrolidine	1.03 ± 0.42 μM	4.24 ± 0.45
32	4A7C-605	Regiomer-1	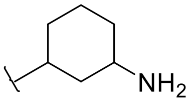	Me	Morpholine	1.08 ± 0.37 μM	5.71 ± 0.81
33	4A7C-606	Regiomer-2	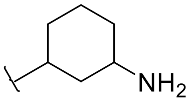	Me	Thiomorpholine	1.54 ± 0.36 μM	5.72 ± 0.16
34	4A7C-607	Regiomer-2	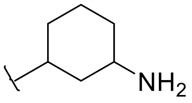	Me	N-Me Piperazine	2.23 ± 0.40 μM	4.50 ± 1.59
35	4A7C-608	Regiomer-2	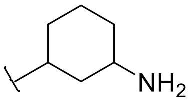	Me	N-Ph Piperazine	1.14±0.13μM	6.31 ± 0.82
36	4A7C-609	Regiomer-2	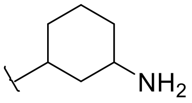	Me	Piperidine	1.19 ± 0.61 μM	4.83 ± 0.51

Supplementary Table 2. Primer sequences for site-directed mutagenesis for generation of constructs. The underlined nucleotide in the sequences of primers used to perform site-directed mutagenesis are the 'mutated' bases.

Primer name	Primer sequences (5'-3')
mNurr-S441A_S	CAGGACCTGCTTTTTGAAGCAGCTTTCTTAGAATTATTTG
mNurr-S441A_A	CAAATAATTCTAAGAAAGCTGCTTCAAAAAGCAGGTCCTG
mNurr-I573A_S	TGCACACAGGGCCTCCAGCGCGCTTTCTACCTGAAATTGGAAGAC
mNurr-I573A_A	GTCTTCCAATTTCAGGTAGAAAGCGCGCTGGAGGCCCTGTGTGCA
mNurr-A586F_S	GAAGACTTGGTACCACCACCATTTATAATTGACAAACTTTTCCTG
mNurr-A586F_A	CAGGAAAAGTTTGTCAATTATAAATGGTGGTGGTACCAAGTCTTC
mNurr-I588A_S	GTACCACCACCAGCAATAGCTGACAAACTTTTCCTGGAC
mNurr-I588A_A	GTCCAGGAAAAGTTTGTCAGCTATTGCTGGTGGTGGTAC
mNurr-K590A_S	CCACCACCAGCAATAATTGACGCACTTTTCCTGGACACCTTACCT
mNurr-K590A_A	AGGTAAGGTGTCCAGGAAAAGTGCGTCAATTATTGCTGGTGGTGG
mNurr-L593A_S	GCAATAATTGACAAACTTTTCGCGGACACCTTACCT TTCTAAACG
mNurr-L593A_A	CGTTTAGAAAGGTAAGGTGTCCGCGAAAAGTTTGTCAATTATTGC
mNurr-D594A_S	ATAATTGACAAACTTTTCCTGGCCACCTTACCT TTCTAAACGCGT
mNurr-D594A_A	ACGCGTTTAGAAAGGTAAGGTGGCCAGGAAAAGTTTGTCAATTAT
mNurr-T595A_S	GACAAACTTTTCCTGGACGCCTTACCTTTCTAAACGCGTGAT
mNurr-T595A_A	ATCACGCGTTTAGAAAGGTAAGGCGTCCAGGAAAAGTTTGTC
mNurr-L596A_S	GACAAACTTTTCCTGGACACCGCACCTTTCTAAACGCGTGATCAG
mNurr-L596A_A	CTGATCACGCGTTTAGAAAGGTGCGGTGTCCAGGAAAAGTTTGTC
mNurr-F598A_S	CTTTTCCTGGACACCTTACCTGCCTAAACGCGTGATCAGCTGTTC
mNurr-F598A_A	GAACAGCTGATCACGCGTTTAGGCAGGTAAGGTGTCCAGGAAAAG
